# p53, p63 and p73 in the wonderland of *S. cerevisiae*

**DOI:** 10.18632/oncotarget.18506

**Published:** 2017-06-16

**Authors:** Olivier Billant, Marc Blondel, Cécile Voisset

**Affiliations:** ^1^ Inserm UMR 1078, Université de Bretagne Occidentale, Faculté de Médecine et des Sciences de la Santé, Etablissement Français du Sang (EFS) Bretagne, CHRU Brest, Hôpital Morvan, Laboratoire de Génétique Moléculaire, Brest, France

**Keywords:** p53, p63, p73, yeast, FASAY

## Abstract

Since its discovery in 1979, p53 has been on the forefront of cancer research. It is considered a master gene of cancer suppression and is found mutated in around 50% of all human tumors. In addition, the progressive identification of p53-related transcription factors p63 and p73 as well as their multiple isoforms have added further layers of complexity to an already dense network. Among the numerous models used to unravel the p53 family mysteries, *S. cerevisiae* has been particularly useful. This seemingly naive model allows the expression of a functional human p53 and thus the assessment of p53 intrinsic transcriptional activity. The aim of this article is to review the various contributions that the budding yeast has made to the understanding of p53, p63 and p73 biology and to envision new possible directions for yeast-based assays in the field of cancer as well as other p53-family-related diseases.

## INTRODUCTION

**Figure 1:**
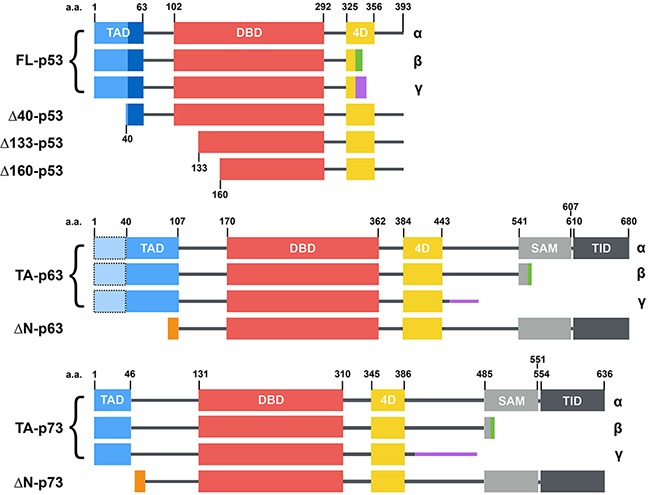
Structure of the main p53 family isoforms (from [32]) p53, p63 and p73 isoforms are generated through secondary initiation codons (Δ40, Δ133 or Δ160) or alternate splicing sites (α, β, γ, etc... C-termini) leading to multiple combinations. p63 and p73 present several other N-terminal and C-terminal isoforms that are not depicted here. p53, p63 and p73 share a similar modular organization with one or two transcription activation domains (TAD), a DNA binding domain (DBD), a tetramerization domain (4D) and two domains specific to p63 and p73: a sterile alpha motif (SAM) and a transcription inhibition domain (TID).

Mutation of p53 is the most common genetic alteration in human cancers [1, 2], making investigations on this tumor suppressor one of the major topics of cancer research [3]. The progressive identification and characterization of p53 mutations led to the description of seven mutational hotspots, which are most frequently found in tumors [4]. In addition, the identification of p63 [5] and p73 [6], two tumor suppressor genes that are related to p53 and of a galaxy of isoforms encoded by p53, p63 and p73 genes has considerably enriched an already vast network [7] (Figure 1). Numerous models have been used to explore the never-ending facets of the p53 family. Among them, the budding yeast *Saccharomyces cerevisiae* has proven to be a precious tool to unravel its mysteries. 37 years after p53 original discovery, this article reviews the various uses of *S. cerevisiae* and envisions new possible applications for already existing yeast-based assays, as well as the creation of original yeast models dedicated to the study of the p53 family.

## IDENTIFICATION AND CHARACTERIZATION OF P53 MUTANTS

The study of p53 role in cancer began with the recognition of its tumor suppressor gene status by the scientific community. Initially cloned in a mutated form from cancerous cell lines, p53 was therefore thought to be an oncogene at first before being instated as a tumor suppressor gene. *S*. *cerevisiae* took part in the early identification of p53 function. In order to verify the suspected transcription activation role of p53, R.W. O’Rourke et al. tested its ability to activate the transcription of the CAT (chloramphenicol acetyltransferase) reporter gene. The amino-terminal fragment of p53 was indeed able to induce transcription and p53 was thus proposed to be a transcription factor of the tumor suppressive response [8]. Furthermore, due to the high degree of conservation of the transcriptional machinery from yeast to human (for review see [9]), E. Schärer et R. Iggo found in 1992 that p53 functions as a sequence-specific transcription factor in yeast [10]. They designed an artificial reporter promoter constituted of a mammalian consensus p53 response element (p53-RE) of 33 base pairs coupled to the weak yeast *CYC1* promoter deleted of its UAS (upstream activating sequences), hence termed “minimal” *CYC1* promoter or mini-*CYC1*. This p53-RE-mini-*CYC1* promoter controls the expression of the *LacZ* reporter gene and therefore allows the detection of p53 transcriptional activity in yeast. Using this system, wild-type p53 (p53-WT) was proven to be a functional transcription factor in yeast. In contrast, some mutants identified in Li-Fraumeni patients’ tumors such as R175H, R248W and R273H were transcriptionally inactive. Hence, the first stone of the path toward a link between mutations of p53 and the loss of its transcription factor activity was laid by the initial characterization in yeast of these three mutations that were not considered hotspot yet. In addition, this work set the ground for the development of FASAY (Functional Analysis of Separated Alleles of p53 in Yeast), which will be described in the next section of this review.

## TOOLS TO STUDY P53 TRANSCRIPTIONAL ACTIVITY

### First generation FASAY

**Figure 2:**
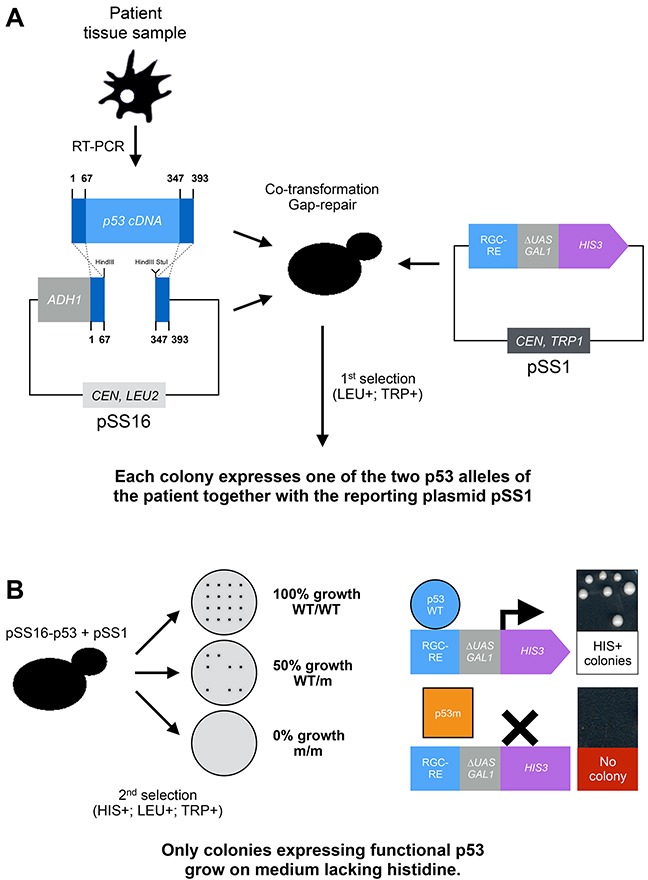
Principle of the FASAY FASAY relies both on the ability of p53 to act as a transcription factor when heterologously expressed in yeast and on the efficiency of homologous recombination in this organism. Indeed, yeast is able to recombine a linear DNA sequence or “target sequence” (*e.g*. PCR product) with a linearized plasmid, which extremities are homologous to that of the target sequence, thereby leading to the “cloning” of the target sequence into the plasmid. This rather efficient natural process is called gap-repair and participates to DNA repair in yeast cells. Here it allows skipping a time-consuming *in vitro* cloning step. **(A)** After obtaining a full p53 cDNA by reverse-transcription from patient fibroblasts, blood or cancerous tissues, the DNA sample is amplified by PCR and introduced into yeast cells together with a linearized cloning plasmid (pSS16) and a reporter plasmid (pSS1). The cloning plasmid pSS16 (that contains *LEU2* gene as a selection marker) contains p53 cDNA deprived of its DNA binding domain leaving only its N- and C-terminal sequences that are required for recombination. The reporter plasmid pSS1 (that contains *TRP1* gene as a selection marker) is constituted of the *HIS3* reporter gene placed under the control the RGC (ribosomal gene cluster) p53 response element fused to the *GAL1* promoter deprived of its UAS (“*mini-GAL1”*). A first selection is made for cells containing both plasmids on a solid medium lacking tryptophan and leucine. These cells express a patient-derived p53 due to the recombination of the p53 cDNA with the pSS16 cloning plasmid *via* gap repair. Due to the frequency of the initial homologous recombination event and the limited copy number of the cloning vector (pSS16), each yeast cell/colony is expected to express a single p53 allele of the patient, hence the name of the assay. **(B)** The second selection is based on the activation of the *HIS3* reporter system. A functional p53 binds to the RGC-RE and activates the *mini-GAL1* promoter, which leads to the transcription of the *HIS3* gene from the pSS1 plasmid. Cells expressing a functional p53 are thus able to grow on a solid medium lacking histidine whereas cells expressing a non-functional allele of p53 are not. The functional status of p53 of a given sample is obtained by analyzing the percentage of cells that are able to form colonies on the medium lacking histidine, leucine and tryptophan compared to the medium lacking tryptophan and leucine: 100% of growing cells indicates a WT/WT status, 50% a WT/m status and 0% a m/m status. WT: Wild-type allele, m: mutated allele.

It soon became clear that p53 mutations were not only a frequent alteration found in human cancers, but also that they were extremely diverse rendering the development of a pertinent test complex and expensive, especially due to sequencing costs at the time. Fortunately, the results published by E. Schärer and R. Iggo in 1992 and discussed above brought the proof of concept of a functional assay to monitor p53 transcriptional activity in this unicellular eukaryote. FASAY was hence developed to assess p53 functionality directly from tissue samples using a *HIS3*-based reporter system (Figure 2) [10–13].

### Colorimetric FASAY

**Figure 3:**
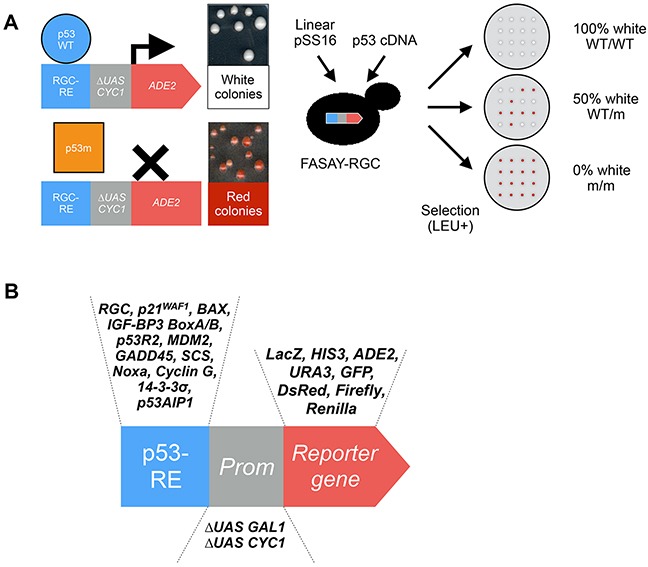
Principle of the colorimetric FASAY and evolution of the FASAY reporter system **(A)** The yeast *ADE2* gene encodes the phosphoribosyl-amino-imidazole carboxylase (Ade2p) enzyme involved in the purine biosynthesis pathway. Its absence interrupts the pathway and leads to the accumulation of the precursor P-ribosyl-amino-imidazole (AIR), which turns red upon oxidation, hence leading to the formation of red yeast colonies. On the contrary, when the pathway is functional due to the expression of a sufficient level of Ade2p, yeast colonies grow white. This reporter system is semi-quantitative as any intermediate level of Ade2p leads to pink colonies whose color intensity is proportional to the level of Ade2p. In the colorimetric FASAY-RGC strain (ylG397), the reporter system has been integrated into the genome of an *ade2*Δ yeast strain thereby limiting the number of selection steps. Here the *ADE2* gene is placed under the control of a promoter composed of 3 copies of the p53 response element RGC (3xRGC) fused to a *mini-CYC1* promoter. FASAY-RGC colonies grow red since no yeast endogenous transcription factor is able to induce transcription from p53 RGC response element. The expression of a functional p53 leads to the production of an amount of Ade2p sufficient to induce the formation of white colonies, whereas non-functional forms of p53 (*e.g*. loss-of-function mutants) lead to red colonies. Intermediate amounts of Ade2p lead to pink colonies and indicate a partial transcriptional activity of the tested p53. A PCR-amplified p53 cDNA and a linearized pSS16 are transformed into FASAY strains. Cells containing a gap-repaired pSS16-p53 are then selected on LEU- medium leading to the growth of red or white colonies. The determination of the “p53 status” of a sample can thus be tested in this single step of transformation by analyzing the percentage of red colonies: 100% of white colonies indicates a WT/WT status, 50% a WT/m status and 0% a m/m status. **(B)** Evolution of the FASAY reporter systems. The reporter system of FASAY has undergone various evolutions using different response elements, promoters and reporter genes.

The FASAY was improved in 1995 by the group of J.M. Flaman [14]. The *HIS3*-based reporter system that relies on a growth/no-growth phenotype was replaced by an *ADE2*-based system in which the lack of expression of the *ADE2* gene can be monitored by a convenient red color phenotype (Figure 3A) [15]. The colorimetric FASAY was validated using p53-WT (97% of white colonies) and a panel of mutants already identified in cancers that were all found inactive (100% of red colonies). Then FASAY was used to determine the p53 status of 21 patients (11 with various cancers, 10 with head and neck squamous cell carcinoma, HNSCC) and turned out to be reliable.

The main cause of background (visualized as a small percentage of red colonies appearing on WT/WT plates) in FASAY resides in the PCR amplification step of p53 cDNA that can lead to 3-18% of *de novo* mutations depending on the fidelity of the polymerase used. The use of a proofreading enzyme as well as mRNA samples of sufficient quality is required in order to obtain satisfying results. Of note, J.M. Flaman et al. used FASAY as a tool to control the quality of PCR enzymes by determining the level of background induced from the amplification of p53-WT [16]. To a lesser extent, false-positives can be due to the gap repair process, which is not 100% accurate or to the co-existence of inactive isoforms of p53 generated by alternative splicing. Later, in order to reduce the background, a version of FASAY called “split-assay” was developed that tested separately the 3′ and 5′ parts of p53 cDNA. By doing so, the localization of mutations can be roughly identified and also differentiated from PCR induced mutations. However, due to the additional steps required, its use has been quite limited [17]. The use of colorimetric FASAY however shows some limitations to detect transcriptional defects of p53 mutants or to compare different response elements since the colorimetric readout is not fully proportional to the transcriptional activity of p53. Indeed partially active mutants and p53-WT can both lead to a white phenotype. Hence, more quantitative versions of the assay based on luciferase or fluorescent reporters were developed. Over time, FASAY underwent several adaptations and evolutions that will be detailed in the following sections of this review (Figure 3B).

## CHARACTERIZATION OF P53 MUTANTS

### Loss-of-function mutants

**Figure 4:**
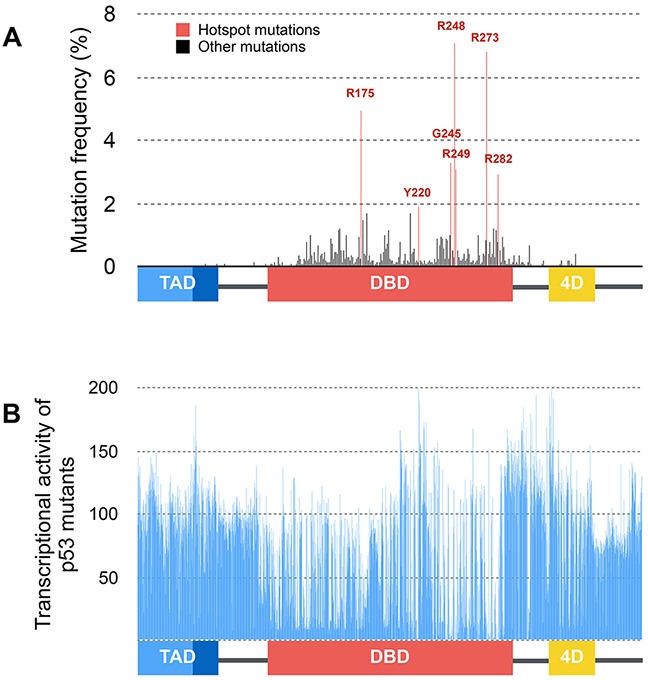
Distribution and functional consequences of p53 mutations **(A)** Distribution and frequency of p53 mutations described in human tumors (data from the International Agency for Research on Cancer). Hotspot mutations are shown in red and are all located within the DNA binding domain of p53. **(B)** Average transcriptional activity of tumor-derived and artificial mutations of p53 on several p53-response elements (p21^WAF1^, Mdm2, BAX, 14-3-3, AIP1, GADD45, Noxa, p53R2) expressed as the percentage of p53-WT transcriptional activity (data from [19]).

FASAY rapidly led to the identification of loss-of-function mutants of p53 among which most of hotspot mutations R175H, G245S, R248Q/W, R249S and R273H (Figure 4A). Although a vast majority of p53 mutations is located in its DNA binding domain, the consequence of mutations outside this zone remained unclear. Using the first generation FASAY, C. Ishioka et al. demonstrated that artificial mutations located in the tetramerization domain (*e.g*. L344P) abrogate p53 transcriptional activity. Also, deletion of the basic C-terminal domain (G354stop) was reported to increase p53 transcriptional activity suggesting that this region exerts an inhibitory effect on p53 [18]. The largest study to date concerning the transcriptional activity of p53 mutants was led by S. Kato et al. in 2003 by adapting FASAY to high-throughput fluorescence-based screening [19]. All possible p53 missense mutants (2,314 mutants) were generated and tested against eight p53-REs. 56% of these p53 mutants are more or less functional which further emphasize the interest of performing a functional assay for p53 rather than a simple systematic sequencing (Figure 4B). Importantly, loss of function is essentially due to the mutation of residues supporting the transcription factor overall structure (core-domain, DNA binding surfaces, secondary structures) and are most frequently found in cancers. These results also confirm that mutations located in the tetramerization domain are likely to impair p53 transcriptional activity. This work was completed in 2004 by the search for p53 temperature-sensitive (ts) mutants using the same system. ts mutations of p53 are mostly located in β sheets of the DNA binding domain and account for 10% of p53 mutations reported in tumors [20].

### Transcriptionally altered mutants

As stated above, the first generation FASAY relies only on the RGC response element (Figure 2 and Figure 3A). Hence, the colorimetric FASAY was enriched in 1998 by the creation of the new FASAY-p21 and FASAY-BAX strains that rely on p53-REs involved in cell cycle control (p21) and apoptosis pathways (BAX) [21] (Figure 3B). In these strains, one copy of p21-RE or one to four copies of BAX-RE (referred to as BAX or BAX4 respectively) have been integrated into the yeast genome. With this panel of FASAY strains, mutations that specifically affect p53 transcriptional activity on a subset of promoters can be detected. Indeed, mutants K120R, R175P, R181H, I254F and R283H were found to activate p21 but not BAX, whereas mutants R175C, R175L, R175S and R181L activate p21 and BAX4. Cell line-derived mutants V272L and H214R give pink colonies indicating a partial transcriptional activity on p21 and were described earlier by the same authors as temperature sensitive mutants as they show a significant transcriptional activity at 25°C but not at 37°C in yeast [14].

From there on, FASAY started being also used as a tool to explore the impact of p53 mutations and additional reporter plasmids were created by Carol Prives's group that include several other p53 target sequences (SCS, Mdm2, GADD45, Cyclin G, IGF-BP3 BoxA/B). Hotspot mutants (R175H, G245D, R248W, R249S R273H and R282A) were found to be transcriptionally inactive on all response elements whatever the temperature used (24°C, 30°C and 37°C) [22]. However, some tumor-derived mutants previously found to be inactive happen to retain some transcriptional activity depending on the p53-RE (P177L, R267W, C277Y and R283H) or on the temperature (V143A, M160I/A161T, H193R, Y220C and I245F) used [22]. Therefore, FASAY allows the identification of p53 mutations that can either abrogate totally or partially its transcriptional activity and/or alter its target spectrum.

### Second-site mutations reactivating mutant p53

Reactivation of loss-of-function mutants could be a pertinent way to fight tumor progression. R.K. Brachman et al. set up a genetic screen in yeast aiming at identifying p53 mutations capable of restoring the transcriptional activity of V143A, G245D, G245S, R248W and R249S mutants. An alternate FASAY has been developed that, instead of *HIS3* or *ADE2*, relies on the *URA3*/5-FOA reporter system that allows both positive and negative selections. Indeed, the *URA3* gene encodes the ODCase (Orotidine 5′-phosphate decarboxylase), which allows yeast cells to grow on medium lacking uracile. But ODCase is also able to convert 5-FOA (5-fluoroorotic Acid) into the toxic compound 5-fluorouracil, thereby preventing yeast cells expressing *URA3* from growing on medium containing 5-FOA [23]. In this alternate FASAY, the *URA3* gene was placed under the control of a p53-RE and led to the identification of second-site mutations T123P, T123A, H168R, S240N and N268D that are able to restore a significant transcriptional activity of several p53 loss-of-function mutations (V143A, G245S, R249S) [24].

### Super-active p53 mutants

Although loss-of-function mutants of p53 gather much of the attention, super-active mutants also play their part in tumorigenesis. Therefore another p53 assay termed “rheostatable FASAY” was developed to identify such mutations [25]. By placing the expression of p53 under the control of the galactose-inducible *GAL1* promoter, finely tunable levels of p53 can be expressed in yeast. This led to the identification of “supertrans” p53 mutants (T123A, S240N, H178Y and V274A), which are stronger transcriptional activators than p53-WT when expressed at a similar level [25]. These mutants of p53 also exhibit target sequence specificities thus potentially leading to highly variable effects in tumors [25–27].

### Dominant-negative mutations of p53

During tumor development, p53-WT transcriptional activity can be neutralized by a dominant-negative effect exerted by mutant alleles of p53 co-expressed in the same cell as a result of heterozygosity. In order to identify dominant-negative mutants, R.K. Brachman et al. developed a FASAY based on the *URA3*/5-FOA reporter system. p53 mutants are co-expressed with p53-WT in yeast. p53 mutants can be considered as dominant-negative if they interfere with p53-WT activity and impede the expression of *URA3*. Dominant-negative mutants were thereby isolated on the basis of their ability to render yeast cells resistant to 5-FOA. However, this system is flawed by a high background of false-positive clones due to spontaneous mutations in the *URA3* reporter gene (87%). Mutations identified as dominant-negative correspond to mutational hotspots (G245S, R248Q, R249S, R273H and R282W) involved in the stabilization of DNA binding surface and DNA interaction. Thereby, the selection of these mutations in cancers could be related to their dominant-negative potential as they lead to a strong, but not total, inhibition of p53-WT activity in yeast [28]. These results were confirmed using the colorimetric FASAY [29–32]. Other mutations such as R156H, H178P, H179R, R181P have been shown to exert a dominant-negative effect whereas hotspot mutations Y220C and R282W were sometimes considered as recessive depending on the cut-off that was chosen [30].

In addition, some mutations retain partial trans-criptional activity while exhibiting a dominant-negative effect toward p53-WT. Indeed, mutants presenting only a partial loss of transcriptional activity toward a specific target sequence may interfere with p53-WT ability to activate the transcription from the same sequence. On this ground, P. Monti et al. established a link between the loss of transcriptional activity of various p53 mutants and their dominant-negative effect [33]. Of note, certain “supertrans” mutants such as T123A and V274A are not affected by the dominant-negative effect of loss-of-function mutants of p53 [25].

## FASAY IN DIAGNOSIS

FASAY stands out as a remarkably efficient tool to identify mutations that interfere with p53 functions as it allows the rapid analysis of multiple samples and limits the sequencing costs. Indeed, only loss-of-function clones were sequenced. FASAY was originally developed in order to detect loss-of-function germinal mutations and, as such, was used in the diagnosis of Li-Fraumeni Syndrome (LFS) and Li-Fraumeni Like syndrome (LFL) [11]. Data obtained from these analyses revealed that mutations of p53 that retain some transcriptional activity are associated with a milder family history of cancer development, a lower number of tumors and a delayed disease onset [34]. Owing to the growing place taken by p53 in cancer research, FASAY has been used to determine the p53 status of various human tumors such as leukemia [35], hepatocellular carcinoma [36] and myelodysplastic syndromes [37] (see [38] for review). FASAY also allowed to determine the dominant-negative potential of p53 mutations in various tumors [30, 31, 39] and helped characterizing the p53 status of various cancer cell lines [14]. The largest study regarding that matter focused on 142 cell lines and revealed that 70% were homozygotes for loss-of-function p53 mutations, 28% were homozygotes for functional forms of p53 whereas only 2% were WT/m heterozygotes [40]. However, the frequency of loss of heterozygosity after immortalization is higher than the one of developing tumors, thus contributing to explain the underrepresentation of WT/m cell lines [40]. Surprisingly, data regarding p53 loss of heterozygosity in tumors remain limited so far [41].

As the interest toward “p53 status” keeps increasing, the reliability, cost-effectiveness and rapidity of FASAY advocates in its favor. FASAY appears even better suited than immunohistochemistry. Indeed, immunochemistry relies on the excessive accumulation of mutant p53 protein, a phenomenon that is far from being systematic and varies depending on the considered mutation [42]. However, no current technology is able to identify all p53 alterations in cancers and FASAY does not permit the identification of the incriminated mutation. Thereby modern sequencing technologies appear to be the perfect companion for FASAY as suggested by R. Iggo et al. [43].

## REACHING OUT TO P63 AND P73

Given that the homology of amino acid sequences between p53 and p63/p73 is around 30% overall and as much as 60% when it comes to the DNA binding domain, it was expected that they share common target sequences. C. Di Como and C. Prives thus used a first generation FASAY to test the transcriptional activity of p73α and p73β isoforms on several p53 response elements (p21, mdm2, GADD45, cyclin G, Bax, IGF-BP3 box A/B, RGC, SCS). They found that these two isoforms of p73 are functional in yeast but exhibit significant variations in their transactivation potential. p73-R292H mutation, which is equivalent to p53-R273H, induces a complete loss of function of both p73α and p73β isoforms. They also reported that, in mammalian cells, p73α physically interacts with p53-R175H and p53-R248Q mutants but not with p53-WT. However they were unable to confirm these results in yeast cells [44]. Such interactions could explain the ability of some p53 mutants to exert a dominant-negative effect over p73. P. Monti et al. pursued the exploration of cross-dominance using colorimetric FASAY. Forty-one mutations (including 4 hotspot and 25 tumor-derived mutations) distributed across all p53 domains were tested and a high percentage of them turned out to interfere with p73β transcriptional activity [45].

More recently, the transcriptional activity of TA/ΔN-p63α and TA/ΔN-p63β isoforms (Figure 1) was tested on 80 response elements in yeast. These isoforms showed great variations in their transactivation potential and in their sequence specificity. TA-p63α demonstrates higher transactivation potential only toward high-affinity elements whereas ΔN-p63α demonstrates an overall lower transactivation potential but targets a wider range of response elements including low-affinity elements. However, these variations seem limited to p63α isoforms and do not concern p63β isoforms in yeast [46].

**Figure 5:**
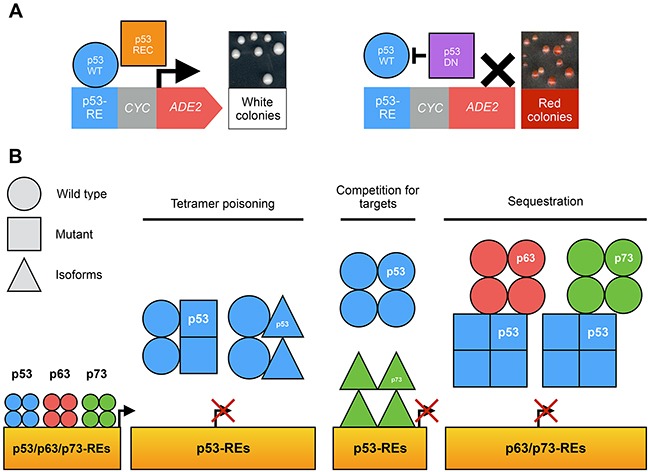
Use of FASAY to study the p53 family **(A)** The co-expression of a functional isoform of p53 family (blue circle) with a recessive isoform or mutant (orange square) leads to the formation of light pink or white colonies. The expression of a functional isoform of p53 family with a dominant-negative isoform or mutant (purple square) leads to the formation of dark pink or red colonies. **(B)** Possible mechanisms of the dominant-negative effect within p53 family.

Our team also relied on the colorimetric FASAY to characterize the dominant-negative extent within the p53 family (Figure 5A) [32]. All loss-of-function hotspot mutants of p53 as well as Δ133-p53α, Δ160-p53α, ΔN-p73α and ΔN-p73β were shown to exhibit various degrees of dominant-negative interference with functional isoforms of p53, p63 and p73. Their dominant-negative effect on p53-WT function relies on the formation of inactive hetero-tetramers between loss-of-function isoforms or mutants of p53 and p53-WT rather than on a prion-like mechanism in yeast, contrary to prior reports [47, 48]. ΔN-p73α and ΔN-p73β are able to interfere with p53-WT function probably by competition for specific binding sites. In addition, we confirmed that p53-R175H interacts with p63 and p73 functional isoforms in yeast. We further showed that this gain of function is stronger when p53-R175H is able to form tetramers suggesting that tetramers of p53 mutants interact with p63 and p73 isoforms [32] (Figure 5B).

Although p63 is scarcely found mutated in cancers, mutations in this gene are strongly linked to developmental syndromes such as ADULT (acro-dermato-ungual-lacrimal-tooth syndrome) and EEC (ectrodactyly, ectodermal dysplasia and cleft lip/palate syndrome) [49]. The major isoform ΔN-p63α as well as TA-p63α are transcriptionally active in yeast contrary to TA*-p63α, which presents 39 additional N-terminal amino acids, suggesting that theses amino acids exert an auto-inhibitory effect. FASAY-derived strains including different response elements (p21, PUMA, Mdm2, BAX, PERP and COL18A1) were also used to monitor the impact of three mutations involved in developmental syndromes (G134V/D, insR155 and R204W) on TA*-p63α, TA-p63α and ΔN-p63α transcriptional activity. G134V/D and insR155 mutations induce a partial loss of p63 transcriptional activity while R204W completely abolishes it. ΔN-p63α-G134V/D and ΔN-p63α-R204W mutants were also shown to exert a dominant-negative effect over ΔN-p63α-WT [50]. In addition, other p53-like mutations of p63, some of which have since been associated with developmental syndromes, were found to severely impair the transcriptional activity of p63γ on p21, Mdm2 and BAX response elements [51].

## P53, P63 AND P73 TARGETS IN YEAST

Baker's yeast allows the expression of a functional human p53 protein, which is isolated from its natural mammalian partners making it a seemingly ideal model to study p53 intrinsic transcription factor activity and the impact of its mutations.

Yeast has also been used to assess the divergence of p53 transcriptional activity throughout evolution. M. Lion et al. compared the transcriptional response of p53 from several species (*Homo sapiens, Mus musculus, Xenopus laevis, Danio rerio, Drosophila melanogaster* and *Caenorhabditis elegans*) using a firefly reporter system under the control of various p53-REs sequences (animal, human and artificial). Significant variations in sequence specificity, temperature sensitivity or transcriptional potential of p53 were highlighted between these different species [52].

In addition, early signs of a transcriptional activity of p53 on yeast endogenous genes have been suggested as p53 induces a growth inhibition in protease deficient strains of *S. pombe* and *S. cerevisiae*. However, p53-dependent yeast cells growth inhibition was also described in *S. cerevisiae* strains that were not protease deficient [53]. In both cases, this phenomenon was triggered by a high level of expression of p53 and suggests that p53 is able to activate the transcription of endogenous yeast genes whose over-expression interfere with cell growth [54, 55]. This hypothesis was further confirmed since loss-of-function mutants of p53 have little or no impact on yeast cell growth when expressed at similarly high level, which suggests that the inhibitory effect of p53-WT is related to its transcriptional activity. The increased toxicity of “supertrans” mutant V122A in yeast further supports this idea but this observation could also be explained by the ability of this mutant to target additional sequences in yeast [56]. In addition, mutations R282W and N268S/I332V lead to a severe growth inhibition in yeast, but this effect is neutralized by the addition of the R337C mutation, which is located in the tetramerization domain [57]. In line with these results, we recently found hotspot mutant p53-R282W to be highly toxic in budding yeast although this mutant is transcriptionally inactive on p53 response elements [32]. The addition of the anti-tetramerization mutation L344P (p53-R282W/L344P) restores yeast growth thus indicating that the oligomerization ability of p53, and therefore potentially its transcriptional activity, is required to interfere with yeast growth [32].

In yeast, the apoptotic machinery seems conserved, since several key elements homologues have been identified that include caspases (*YCA1*), apoptosis-inducing factor (*YNR074C* or *AIF1*), endonuclease G (*NUC1*) or Bcl2-like proteins (Ybh3p) [58]. Confirming the adversary effect of p53 on cell growth, V. Palermo‘s group showed that p53 triggers yeast apoptosis pathway through *NUC1* [58]. Moreover, in *S. cerevisiae*, p53-WT, but not p53-R248W, induces signs of apoptosis which are associated with the repression of thioredoxin genes (TRX1/2) and the production of high levels of reactive oxygen species [59]. In addition p53 seems to target *ACT1* which over-expression is known to be toxic in yeast [60, 61]. Of note, expression-dependent cell growth inhibition in yeast has also been observed for TAp63α, ΔNp63α and TAp73α but seems to involve autophagy rather than apoptosis [62]. p53 has also been shown to interfere with the yeast DNA damage response by reducing intra-chromosomal recombination [63]. The existence of such endogenous p53 targets in yeast also led to the development of new reporter systems [61]. Although the interest to study endogenous p53 family targets *in levuro* may appear limited, it provides interesting elements to the understanding of p53 family transcriptional role. And even if the quest for a p53 orthologue in yeast remains unsuccessful to date, answers may come from digging deeper in the function of the older evolutive member p63, which has been implicated in female germline protection during meiosis in vertebrates [64]. This function is indeed quite similar to that of Ndt80, a yeast transcription factor implicated in nucleolar damage control during meiosis, which shows 14% of identity and 24% of similarity with TA-p63α [65, 66] and is a potential yeast orthologue of p53/p63/73.

## LOOKING FOR CELLULAR PARTNERS

Yeast two-hybrid (Y2H) allows the identification of protein-protein interactions and has been used to explore the vast interaction network of p53. Y2H allowed the identification and characterization of some of p53 most prominent partners like Mdm2. Mdm2 is an E3-ligase which is a core regulator of p53 in charge of maintaining cellular p53 levels low in the absence of stress by promoting its ubiquitination and consequently its degradation by the 26S proteasome. Mdm2 was also found to inhibit p53 transcriptional activity by binding to its transactivation domain [67]. Y2H was later used to look for partners of Mdm2 that would affect p53 function (reviewed in [68]) as well as to explore the p53-Mdm2 interaction in lower eukaryotes such as *M. trossulus* [69]. Two other important regulators of p53, 53BP1 (double strand breaks repair) and 53BP2 (ASPP family), were identified thanks to this yeast-based technique [70]. Several regulators of p53 have been found as well that include TAF3 [71], BAF60 [72], Ki-1/57 [73] or influenza viral membrane protein BM2 [74]. In addition, Y2H has been used to explore the impact of p53 mutations on its interactions with SV40 oncovirus large T antigen [75], 53BP1 [76] or MBP1 [77]. More recently, a high-throughput Y2H screening aiming at mapping all possible human protein-protein interactions led to the identification of multiple new potential partners of p53 [78]. Among those, the unknown gene HSU79303 has been shown to encode the putative coiled-coil domain-containing protein CCDC106 [79].

Due to their late identification, p63 and p73 are not renowned for their large social networks but can be expected to close the gap in a near future. At least two p63 partners have been discovered using Y2H: Stxbp4 [80] and Setdp1 [81]. p73 partners screenings have been more prolific with HPV E6 proteins [82], c-Myc and MM1 [83], RACK1 [84], RanBPM [85], PKA-Cβ [86], p19ras [87] and BCA3 [88]. p63 and p73 have yet to benefit from comprehensive interaction screenings that could help clarifying their network and thus their role in development and cancer.

Interactions within the p53 family have also been subjected to Y2H experiments and showed strong homotypic interactions (p53/p53, p73β/p73β, p63/p63) but much lower heterotypic interactions due to the diversification of their respective tetramerization domains [6, 89]. However, due to their transactivator properties, full-length p53, p63 and p73 cannot be used in Y2H, which limits the pertinence of this assay regarding the determination and study of intra-family interactions.

Yeast three-hybrid, a variant of the yeast two-hybrid, allows the detection of interactions between proteins and RNA. Due to the highly basic composition of the p53 C-terminal domain, K.J.-L. Riley et al. looked for possible RNA sequences able to associate with p53. They showed that p53 is indeed able to bind RNA sequences *via* its C-terminal domain in yeast, although no RNA sequence or structure specificity was identified. Specific p53-RNA interaction might indeed be dependent on post-translational modifications of its C-terminal domain that do not occur in yeast [90].

## YEAST-BASED PHARMACOLOGICAL SCREENING TARGETING p53

The discovery of drugs targeting p53 remains highly dynamic but in spite of multiple promising candidates identified *in vitro*, many have fallen short *in vivo* [91]. Two approaches are essentially developed in order to reactivate p53 function in cancerous cells. The first aims at neutralizing the interaction between p53 and its most potent inhibitors Mdm2 and Mdm4/X, whereas the second aims at reactivating p53 mutants.

Mdm2 interacts with p53 in yeast and induces a diminution of its transcriptional activity. However Mdm2 does not induce a significant degradation of p53 in yeast, but can bind to its transactivation domain. Mdm2 would thus interfere with transcription cofactors such as 53BP1, through a competitive mechanism. These results indicate that, although most of the p53 pathway is absent from yeast [92], the interactions between p53 and its partners remain possible; but this question is still debated [93].

**Figure 6:**
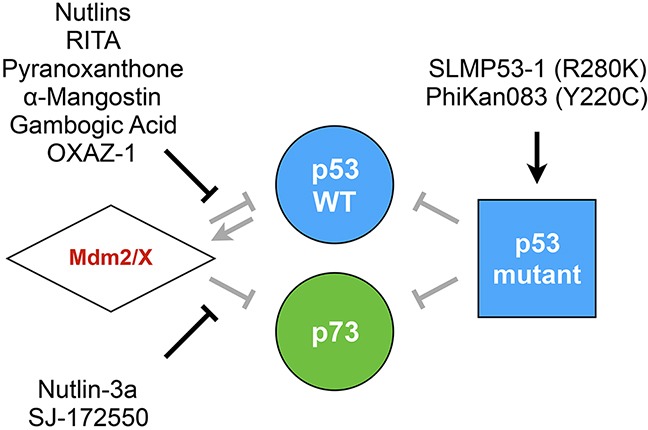
Compounds targeting the p53 family Yeast-based pharmacological screenings led to the identification and/or study of several compounds that are able to restore the p53 family tumor suppression function. Such drugs target the regulation of p53/p73 by Mdm2/X or lead to the restoration of mutant p53 transcriptional activity.

In order to use yeast as a model for pharmacological screening, FASAY has once again been adapted by replacing the *ADE2* reporter gene by Firefly or Renilla luciferase reporters allowing a miniaturization of the assay and hence a high throughput. Compounds targeting the p53-Mdm2 interaction (Nutlin and RITA) are also effective in yeast and restore p53 transcriptional activity, thereby validating the yeast model for further screening. In contrast, PRIMA-1, which restores mutant p53 transcriptional activity *in vitro*, happens to be inactive in yeast [92]. Recently, M. Leão et al. set up a new functional test that relies on p53-induced growth inhibition in yeast. Indeed, when expressed in yeast, Mdm2 interferes with p53 transcriptional activity, which allows cells to grow normally. A compound that disrupts the p53-Mdm2 interaction restores p53 function and thereby limits cell growth. Sixty different xanthone derivatives were screened first *in silico* for their ability to bind Mdm2 and then in yeast for their ability to restore p53-dependent growth inhibition. Several candidate drugs were isolated that include pyranoxanthone that turned out to be also active in mammalian cells [94], α-Mangostin and Gambogic Acid [95]. The same approach was later used to develop a yeast-based screening assay that aimed at targeting the p53-MdmX interaction [61] and led to the identification of OXAZ-1 which is able to prevent the interaction of p53 with both Mdm2 and MdmX [96]. Nutlin-3a which prevents the p73/Mdm2 interaction and SJ-172550 which targets the p73/MdmX interaction were also identified [62]. Unlike p53-WT, certain p53 mutants do not interfere with grow-inhibition, which was used to screen mutant-reactivating drugs. This led to the identification of SLMP53-1, a compound that reactivates the R280K mutant as well as the validation in yeast of the ability of PhiKan083 to reactivate the Y220C mutant (Figure 6) [97].

## CONCLUDING REMARKS

p53 has travelled across numerous models, most of the time with its mammalian companions, and seems to fit in everywhere but it definitely found a second home in baker's yeast. From the early years of p53 characterization to recent high-throughput pharmacological screenings, yeast has proven to be a versatile tool, which helped unraveling p53 mysteries. Now 21 years old, FASAY has undergone various transformations and evolutions to accompany the new questions about p53, p63, p73, their mutants and isoforms and it will probably not stop there as the p53 field is far from drying out. The p53 family remains an “unresolved puzzle” [98] of which many pieces and combinations can be challenged in yeast. Indeed, the different p53, p63 and p73 isoforms exhibit various transcriptional potentials and specificities in yeast [32], but how different isoforms can assemble and what role such chimeras may play remain open questions. The diverse humanized yeast FASAY strains should provide considerable insight into that matter. However p53 family reunions are not always joyful and if the dominant-negative landscape of the p53 family isoforms begins to take shape, the deleterious effect of p53 mutants has yet to come around. Loss-of-function mutants and isoforms of p53 interfere with p53-WT through tetramerization but this could be bypassed using p53-WT proteins harboring an alternate tetramerization domain that would render them insensible to the mutant [99]. In addition, the interaction of mutant p53-R175H with p63/p73 happens through a different mechanism that is likely to be mutant-specific and therefore possibly druggable. As only a few mutants have been characterized regarding their dominant-negative cross-talk capacities, yeast strains recapitulating such interactions may prove themselves pertinent drug screening models in the future. In addition yeast-based drug screenings that aim at identifying p53 protein-protein interaction inhibitors are already giving promising results. But can compounds that restore p53 mutant function in yeast without affecting p53-WT be identified? The fact that “supertrans” mutants identified by genetic screening are apparently able to overcome p53 loss of function induced by dominant-negative p53 mutants strongly suggests that this may be possible. Genetic screenings have been scarcely used for p53 yet, but since the conservation of the apoptotic pathway between yeast and human is being unveiled, they could be used to identify new ways of escaping the deleterious grasp of p53 mutants and isoforms. Native p53 family targets in yeast also represent an unexpected area of development that could help understanding the role of p63 and p73 in meiosis. The emergence of p53 siblings, p63 and p73, has extended the role of these genes to diseases other than cancer. p53-like hotspot mutations of p63 have been found in developmental syndromes and their behavior seems strongly similar to that of p53. Advances in p53 cancer research may thus find even more applications than previously thought. This may well be true for p73 mutations as well, provided that some mutations could be identified and associated to particular diseases or syndromes. Three decades of p53 research using yeast are ready to be applied to p63 and p73 to tackle these other pathologies.

In a sense, p53 really recalls the disease it contributes to cause. In the early developments of chemotherapy, the question asked was: is it possible to destroy cancerous cells while preserving the sane ones? With p53 one can wonder: how can we disable its mutants or isoforms while preserving its functional elements? The answer will not come easily, but baker's yeast is likely to help solving this critical issue.
